# A platform technology of recovery of lactic acid from a fermentation broth of novel substrate *Zizyphus oenophlia*

**DOI:** 10.1007/s13205-014-0240-y

**Published:** 2014-08-22

**Authors:** Moumita Bishai, Swarnalok De, Basudam Adhikari, Rintu Banerjee

**Affiliations:** 1 Microbial Biotechnology and Downstream Processing Laboratory, Agricultural and Food Engineering Department, Indian Institute of Technology, Kharagpur, 721 302 India; 2 Materials Science Centre, Indian Institute of Technology, Kharagpur, 721 302 India

**Keywords:** Lactic acid, Purification, Ion exchange resin, Amberlite IRA 96, Amberlite IR 120

## Abstract

Lactic acid, a biologically derived compound, exists ubiquitously in nature. Its existence ranges from human being to microorganisms. Having paramount industrial significance, lactic acid should be highly pure, devoid of any contaminants. Hence, development of minimum steps of platform technologies to purify it needs urgent attention. The article proposed a novel and simple process for separation of lactic acid from a potential substrate *Zizyphus oenophlia*, based on ion exchange chromatography. The process herein involves two steps of purification; firstly a weak anion exchange resin was used to separate lactic acid from other anions present in the broth. This was followed by use of strong cation exchanger which washes out the target molecule (lactic acid) while trapped other cations present in the solution. The selected ion exchangers were Amberlite IRA 96 and Amberlite IR 120. Amberlite IRA 96 retained the lactic acid from the broth while washing away other anions. Maximum binding capacity of the resin was found to 210.46 mg lactic acid/g bead. After the simple two-step purification process, the purity of lactic acid improves up to 99.17 % with a recovery yield of 98.9 %. Upon characterization, formation of only levo rotatory form of lactic acid confirms its easy metabolism by the human system, thus triggering its application towards biomaterial sector.

## Introduction

Chemical industries are undergoing a transition in their orientation from conventional chemical production to biotechnological production. One of the main aspects of biotechnological process development, which is considered during the production and purification of valuable biomolecules, is tailoring of the bioseparation techniques specifically for isolating the targeted product to impart improved resolution, simplicity, selectivity along with easy scaling up of the process (Tong et al. 2004; Negi et al. 2011). Biotechnologically formed products are known for more than a century and are used in different industrial sectors. Lactic acid is one such product which finds its application in multitude sector. It is produced from varied range of carbohydrate-rich renewable resources using fermentation technology (Wee et al. 2006; Bishai et al. 2013a). It can be metabolized by the human system due to the presence of l-lactate dehydrogenase inside the body which triggers its application in biomaterial sector. l (+) lactic acid is the raw material for the production of polylactic acid (PLA) (Bishai et al. 2013b). To obtain high-quality polylactic acid, l (+) Lactic acid with high purity is the prerequisite. The purification of lactic acid from the aqueous fermentation broth remains a problem considering its purity, recovery and cost of purification.

A variety of different downstream processing methods like precipitation with calcium hydroxide, electrodialysis, crystallization, extraction, etc. have been reviewed recently for purification(Kaufman et al. 1995; Madzingaidzo et al. 2002; Pal et al. 2009; Gonzalez et al. 2007). Most of these separation technologies have either shown low selectivity or low recovery yield. Crystallization for example demonstrated high selectivity, yet, its low recovery of mother liquor restrained its application for large-scale purpose (Caboche et al. 2000). Hence, the propensity for chromatographic techniques rose due to its low cost, high selectivity and ease of operation (Sosa et al. 2000). Among the various categories of chromatographic techniques, ion exchange chromatography for integrative purification has been developed to diminish the overall time required for conventional downstream processing and increase the product yield. Its other advantages include requirement of low cost raw material, execution in shorter time period, moderate operational conditions and generation of minimum amount of wastes (Raya-Tonetti et al. 1998). Considering ion exchange chromatography, several kinds of ion exchangers (Zheng et al. 1996; Chol and Hong 1999; Cao et al. 2002) have been studied on the recovery of lactic acid in the past few years. To successfully design the purification processes, it is of utmost importance to investigate and accordingly adjust the process parameters like selection of the resin, efficiency of binding, adjustment of elution conditions, etc. in a way, to selectively isolate the molecule of interest among all the other contaminants.

The present investigation concentrated on reduction of the steps of purification process, which may lessen its production cost and percentage loss. Two-step separation techniques to purify lactic acid from the fermentation broth including anion and cation exchange chromatography were performed. Amberlite IRA 96 and Amberlite IR 120, containing styrene-divinyl benzene matrix with polyamine functional group and sulfonic acid, respectively, as the active group, were considered for the present study. The binding capacity, breakthrough curve, elution condition, flowthrough and washing condition during purification process for lactic acid were described. Moreover, other parameters in terms of recovery percentage, purity, etc. were also considered for justifying the process.

## Methods

### Fermentation of lactic acid

*Zizyphus oenophlia* was used as a substrate for lactic acid fermentation using *Lactobacillus amylophilus* GV6. Modified de Man, Rogosa and Sharpe (MRS) medium and substrate ratio of 3:2, inoculum volume of 3 % at 35 °C and pH 6.5 for 96 h having 0.7 % CaCO_3_ were optimized conditions for fermentation which resulted in 95.09 % conversion to lactic acid (Bishai et al. 2013a). After fermentation, the characteristics of the fermented broth includes l-isomeric form of lactic acid having concentration of 200 mg/ml, pH 1–2 and residual sugar content 0.86 %.

After fermentation, the fermented broth was centrifuged at 8,000×*g* for 20 min. The supernatant obtained from the extract of fermented broth was used for lactic acid estimation according to the Kimberley and Taylor (1996) method.

The supernatant obtained after centrifugation was subjected to ultrafiltration (Vivascience, USA) using a membrane of 10,000 molecular weight cut off. The permeate portion collected after ultrafiltration was used for purification.

### Ion exchange resin preparation

Amberlite IRA 96 (Sigma Aldrich, USA) and Amberlite IR-120 (Sigma Aldrich, USA), as anion and cation exchangers, respectively, were selected for the particular study. Both the resins comply with the legislation on food product uses. Amberlite IRA 96 is a weakly basic anion exchange resin with an unusually high capacity for large organic acids. Before utilization, the resin was washed and converted into their Cl^−^ form as it could not be obtained in OH^−^ form (resin remained in free base form at pH higher than 9) (John et al. 2008). Resins in Cl^−^ form were obtained by washing the resins sequentially with 1 N HCl solution, distilled water, 1 N NaOH solution, distilled water, 1 N HCl solution and distilled water (until pH 7). Similarly, Amberlite IR-120 was regenerated to H^+^ form by washing with 1 N HCI, and thoroughly rinsed with distilled water (Rincon et al. 2007).

### Effect of pH of fermented broth on lactic acid binding

25 g of resin of IRA 96 and 150 mL of ultrafiltrated lactic acid broth at different initial pHs (2, 3, 4, 5, 6, 7, 8, 9, 10) adjusted with NaOH were taken in a conical flask and kept for shaking. After 12 h, samples of the supernatants were withdrawn and analyzed for lactic acid. pH for maximum lactic acid binding was determined by comparing the percentage of lactic acid from the fermentation broth that had adsorbed to the resin in each case.

### Binding capacity of the resin

In the current study, 25 g of IRA 96 resin and 25 mL of ultrafiltrated lactic acid broth of different concentrations (10, 25, 50, 75, 100, 150, 200, 250 mg lactic acid/mL) were mixed for direct contact in 100 mL flasks at 25 °C and kept in a mechanical shaker. After 12 h, samples of the supernatants were withdrawn and analyzed for lactic acid through HPLC. Residual lactic acid concentrations in the solutions were measured and the amount of lactic acid adsorbed per gram resin was calculated.

### Breakthrough curve determination

A column (length, 20 cm; i.d., 1.6 cm) packed with ~25 g resin was used for column separation. The column was washed with water after the resin was regenerated. After adjustment of desired pH, ultrafiltrated sample of lactic acid was applied on the column at a flow rate of 0.8 mL/min. Eluate was collected and the lactic acid concentration was measured by HPLC.

### Effect of different normality of HCl on the elution profile of lactic acid

In this study, HCl of different normality (0.05, 0.1, 0.25, 0.5, 0.75 and 1 N) was added as eluent to ~25 g of lactic acid loaded resin, packed in ion exchange column. All the fractions containing lactic acid in eluate were pulled together in each case and analyzed for concentration and percentage purity of lactic acid.

### Effect of washing capacity of the cationic resin

Fractions containing lactic acid in the eluate after purification through anion exchange were collected and adjusted at different pH (2, 3, 4, 5, 6, and 7) with NaOH. The individual fractions were fed to the ion exchange column packed with ~25 g of IR 120 resin which is cationic in nature. To remove the contaminations left after first step of ion exchange, this technique of negative chromatography was adapted, where the contaminants were selectively retained in the column and lactic acid is obtained in the flow-through or in the washing steps.

### Analysis of lactic acid through high performance liquid chromatography (HPLC)

Concentration and purity of lactic acid were analyzed by HPLC system with UV–VIS detector at 210 nm. Zorbax C^18^ column (4.6 × 250, 5 μm) was used with a mobile phase of 0.1 M orthophosphoric acid. The flow rate was fixed at 0.75 mL/min and the injection volume was 20 µl. The concentration and percentage purity of lactic acid in eluate were determined from the peak area and number of peaks.

## Characterization of lactic acid

### UV–Vis spectrophotometer

Spectral analysis was performed through spectrophotometer UV–Vis spectrophotometer (Agilent technologies, USA). Purified lactic acid was taken separately in quartz cuvette and absorption spectrum was determined over a range of 205–700 nm wavelengths. The method was performed according to Guo et al. (2012).

### Circular dichroism (CD) spectropolarimeter

Circular dichroism spectropolarimeter JASCO (J-810) with optical activity at 25 °C, wavelength of 210 nm and a concentration of 1 g/dL in chloroform has been used to measure the enantiomeric purity of the samples with excellent precision. The specific optical rotation of purified lactic acid was measured at 25 °C using Circular dichroism spectropolarimeter within a range of 190–280 nm wavelength. l-Acids show a positive whilst the D-isomers show a negative effect in the range of 220–230 nm according to Gargely and Polarimetry (2000). By comparing with standard l-lactic acid, the optical purity of the products was confirmed.

## Results

### Initial purification by centrifugation and ultrafiltration

Centrifugation of the fermented broth was the preliminary step performed prior to the main purification process. The validation and efficiency of the step were observed through the HPLC profile of the supernatant of centrifugation (Fig. 5). Relative purity of lactic acid at this stage was 48.69 % (in terms of peak area) and 1/6 in terms of number of peaks. Removal of cells, cell debris and other insoluble solid residues are performed through this step. The supernatant thus collected was subsequently subjected to Ultrafiltration process. The grossly insoluble solid-free supernatant of fermented broth after centrifugation was fed to ultrafiltration unit fitted with a 10,000 Da molecular weight cutoff membrane. Relative purity of lactic acid did not increase considerably in terms of peak area (56.24 %). However, the concentration of lactic acid in the permeate increased keeping the relative purity (1/6) almost same. This was further validated through the HPLC profile (Fig. 5).

### Purification using ion exchange chromatography

Before actually performing the separation technique, it is very important to get some idea about the process parameters such as pH of the fermented broth, binding capacity of the anionic resin, breakthrough curve, strength of HCl for elution, washing capacity of cationic resin and the effect of contaminants in the fermentation broth on its lactic acid adsorption.

### Effect of pH of fermented broth on lactic acid binding

**Fig. 1:**
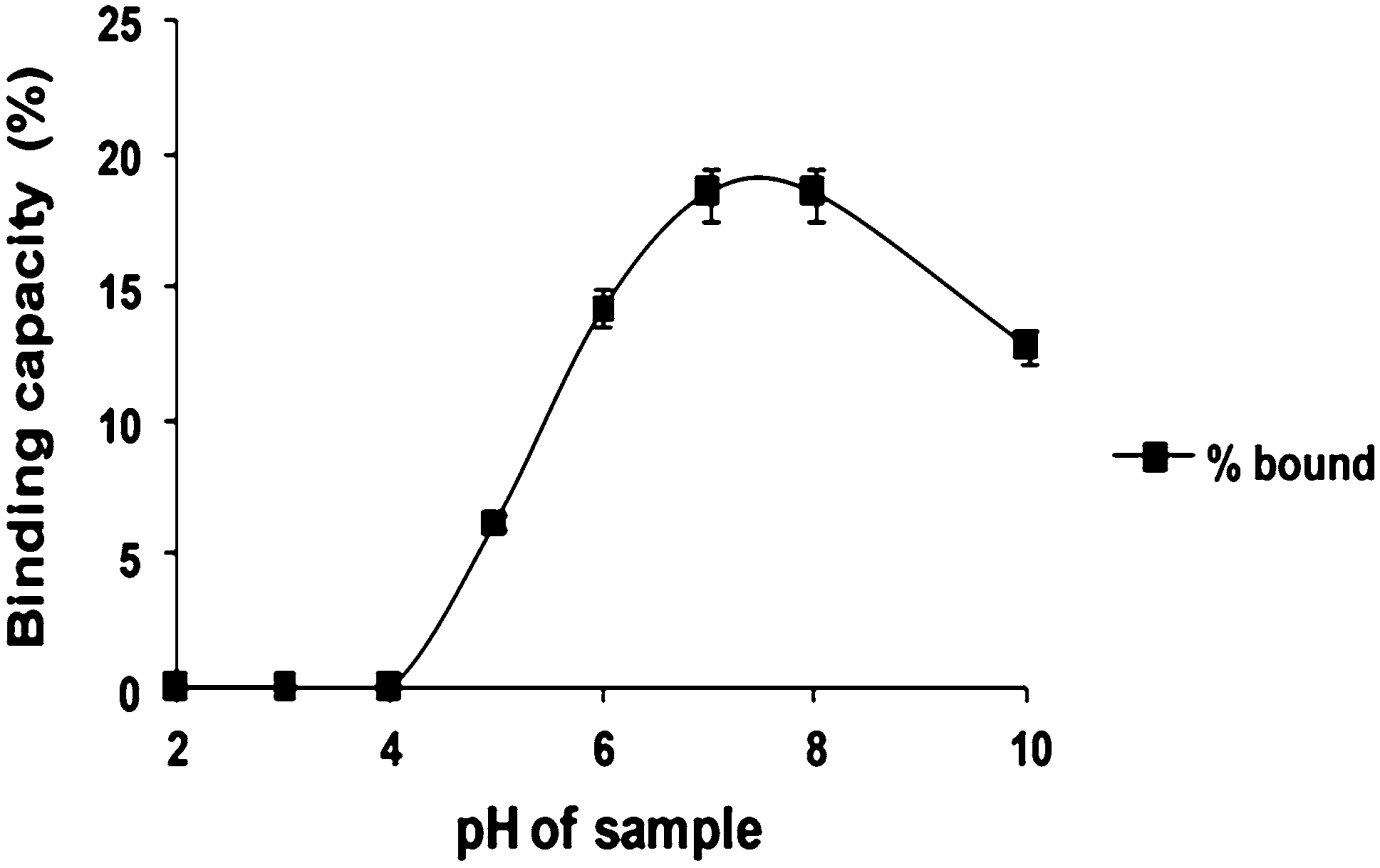
Effect of pH on the binding of lactic acid by Amberlite IRA 96 resin. The sample was shaken for 12 h at the appropriate pH independently

To achieve maximum adsorption efficiency, selection of the pH of the broth before loading it to the column is vital. It was clearly illustrated that initially from pH 2–3 no lactic acid binding occurred (Fig. 1). As the pH increases, absorption also increases. pH 5 showed maximum adsorption where 18.5 % of the total lactic acid content in the fermentation broth (30 g of lactic acid in 150 ml) adsorbed on the beads. Further increase in pH, however, reduced the lactic acid adsorption.

### Binding capacity of the resin

**Fig. 2:**
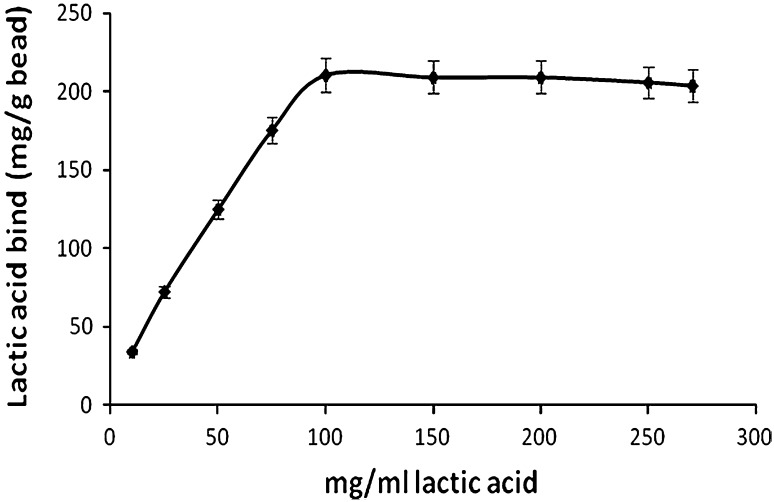
Profile of binding capacity of the Amberlite IRA 96 resin at different concentrations of lactic acid. Lactic acids of appropriate concentrations were shaken for 12 h at 25 °C

The capacity of the resins to adsorb lactic acid was determined using different concentrations of lactic acid solution (Fig. 2). The result indicated gradual increase in lactic acid binding with the increasing concentration of lactic acid in the solution. At 100 mg/mL lactic acid concentration, the resins showed highest binding of lactic acid (210.46 mg lactic acid/g bead). After which as the concentration of lactic acid increased, no significant change was observed in the binding of lactic acid.

### Breakthrough curve

**Fig. 3:**
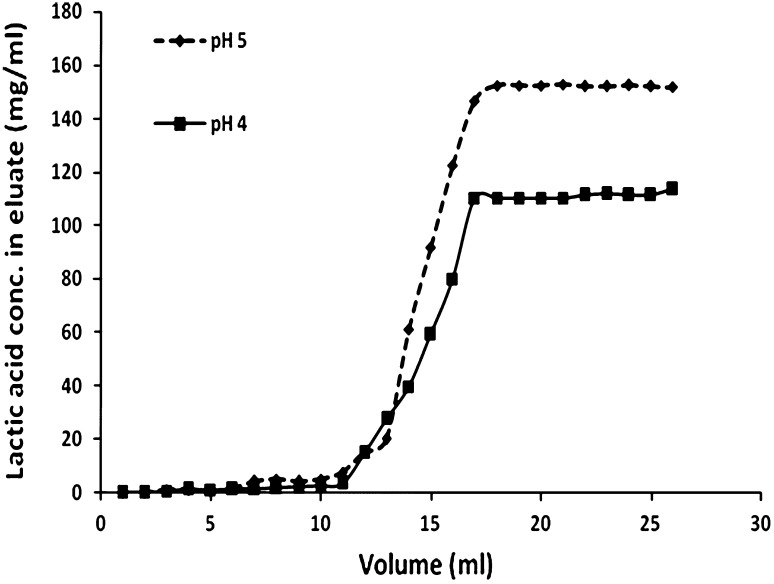
Breakthrough curve at different pH for anion exchange resin Amberlite IRA 96 at a flow rate of 0.8 mL/min. ‘’ represents pH 4, whereas ‘’ represents pH 5

Breakthrough curve provided an initial step for maximizing the adsorption rate until the resin reached saturation; after that, the concentration of the target ion noticeably increased in the effluent. The breakthrough curves of l (+) lactic acid in fermentation broth are shown in Fig. 3. The concentration of l (+) lactic acid in eluate increased rapidly at elution from 15 to 22 mL fraction and reached a plateau after 25 mL. The maximum adsorption capacity shown in Fig. 3 was obtained at pH 5.0 where approximately 200 mg lactic acid bound to 1 g resin. Apart from this, the curves have an S-shape.

### Determination of strength of HCl

**Fig. 4:**
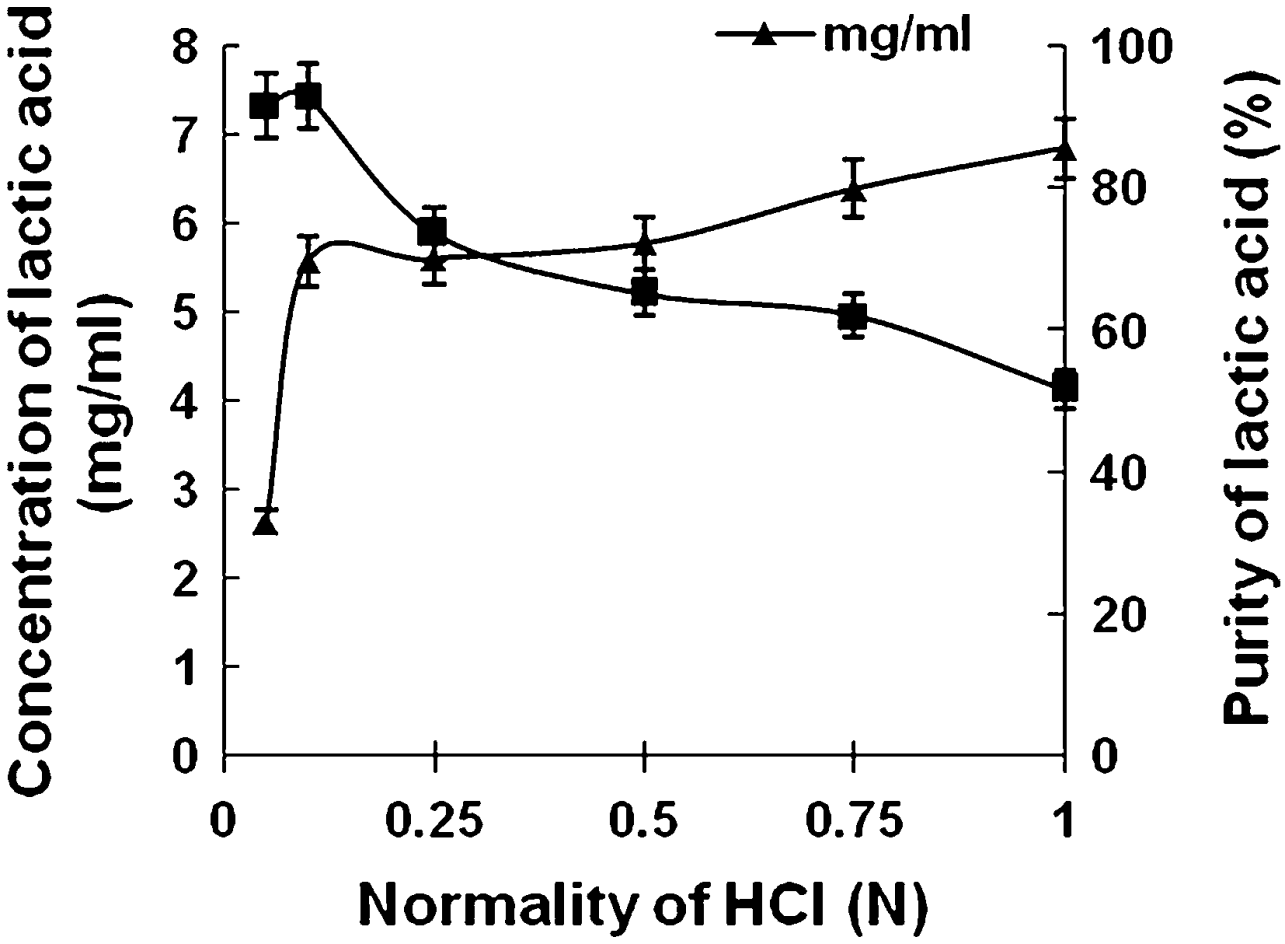
Elution profile of lactic acid adsorbed on IRA-96 at pH 5 by various eluents (HCl of different strength)

Almost complete recovery was achieved with HCl from Amberlite IRA 96. The amount of lactic acid recovered against varying normality of HCl has been determined along with respective purity percentage (Fig. 4). Highest elution was obtained with 0.1 N HCl showing 93.72 % purity of lactic acid which decreases on further increasing the normality of HCl. Increasing HCl strength further showed decrease in purity percentage. Though such enhanced purity is highly encouraging, still for its proposed application in pharmaceutical field, a higher level of purity seemed to be imperative.

### Cation exchange chromatography

**Fig. 5:**
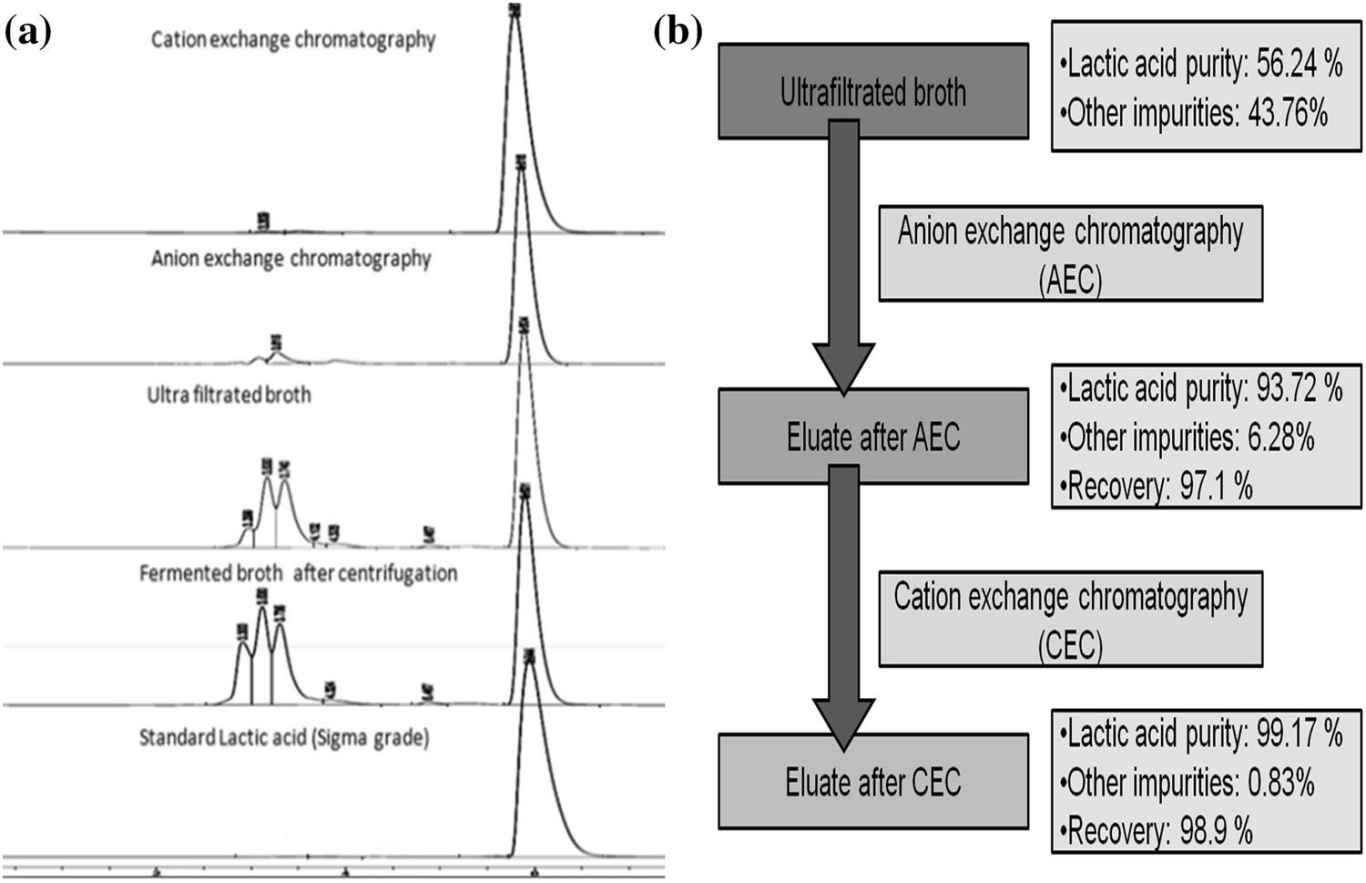
Study on **a** HPLC chromatogram obtained at different purification steps, **b** purity percentage obtained after every step of purification

**Fig. 6:**
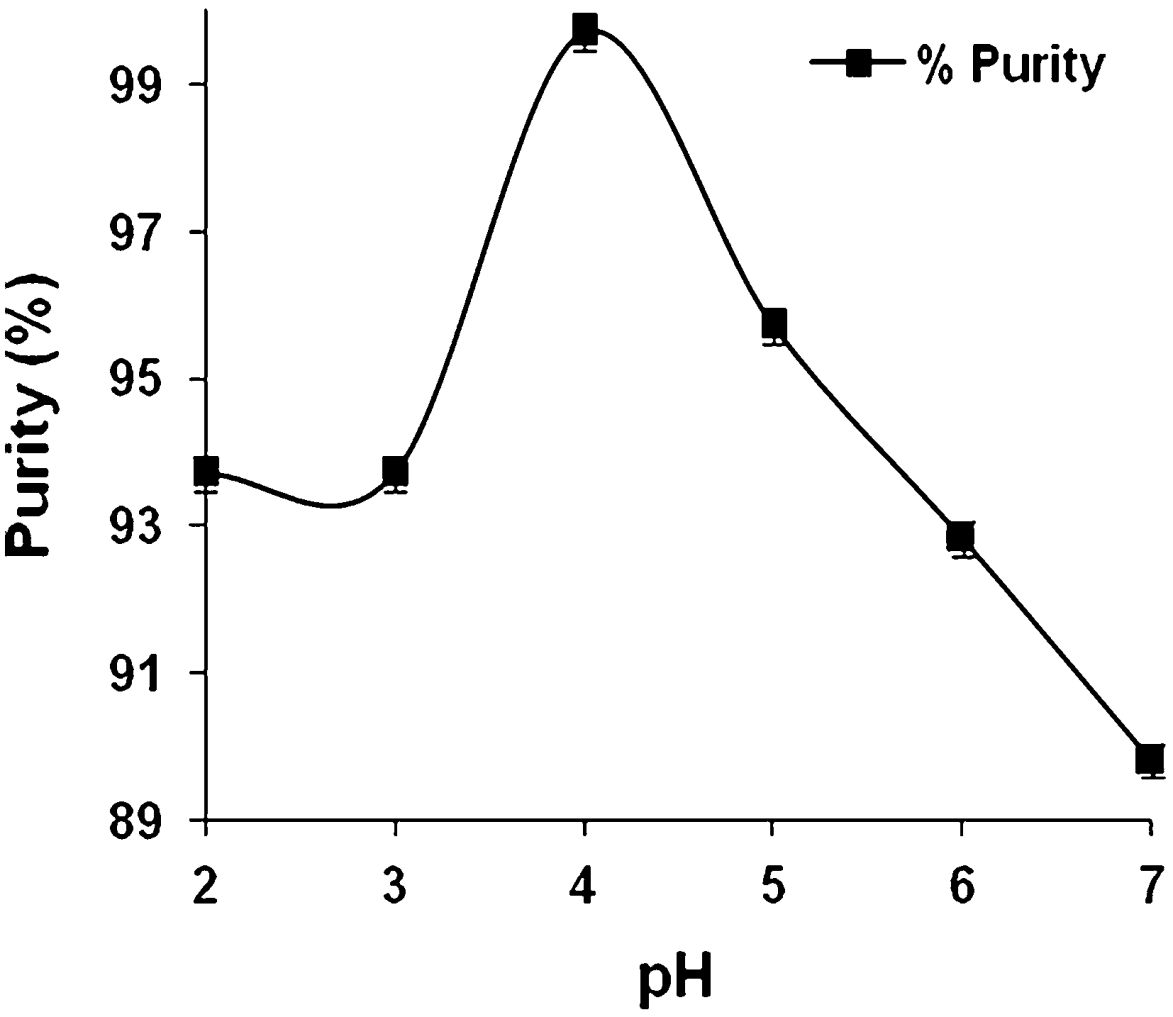
Effect of different pH of fermented broth on washing capacity of cation exchange resin Amberlite IR 120 from pH 2 to 7

Though more than 90 % of purity was obtained with anionic resin from the HPLC profile (Fig. 5), further purification was attempted using cationic capacity of the resin, i.e., Amberlite IR 120. In this case, the washing capacity of the resin was determined at different pH of the eluate sample which showed that at pH 4 the maximum purity of the sample was 99.17 %. Hence, percentage purity increased (Fig. 6) which was also shown in HPLC chromatogram (Fig. 5).

## Characterization of purified lactic acid

### UV–Vis spectrophotometer

**Fig. 7:**
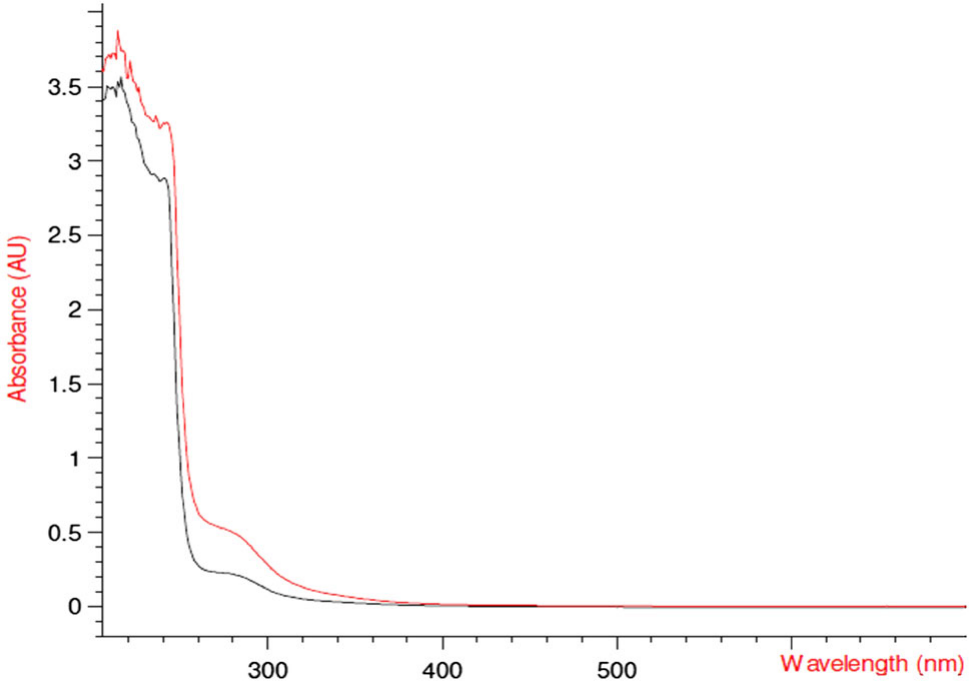
UV–Vis spectrum of the purified lactic acid sample along with the pure sigma graded l-lactic acid. Here *red line* represents the UV scan of purified lactic acid sample and *black line* represents the UV scan of pure sigma graded l-lactic acid

The spectral structures of the UV/VIS absorption indices of lactic acid dissolved in water are very similar. The band observed in the spectra is the weak π* ← *n* transition of the carboxylic group of lactic acid. It is known to start close to ∼300 nm with its maximum around 260 nm in aqueous solutions (Maria and McGlynn 1972; Beavers 2005). In the investigated samples, the bands are slightly displaced due to conjugation, substitution and solvent effects. Figure 7 shows the absorbance in the UV–Visible region.

### Circular dichroism

**Fig. 8:**
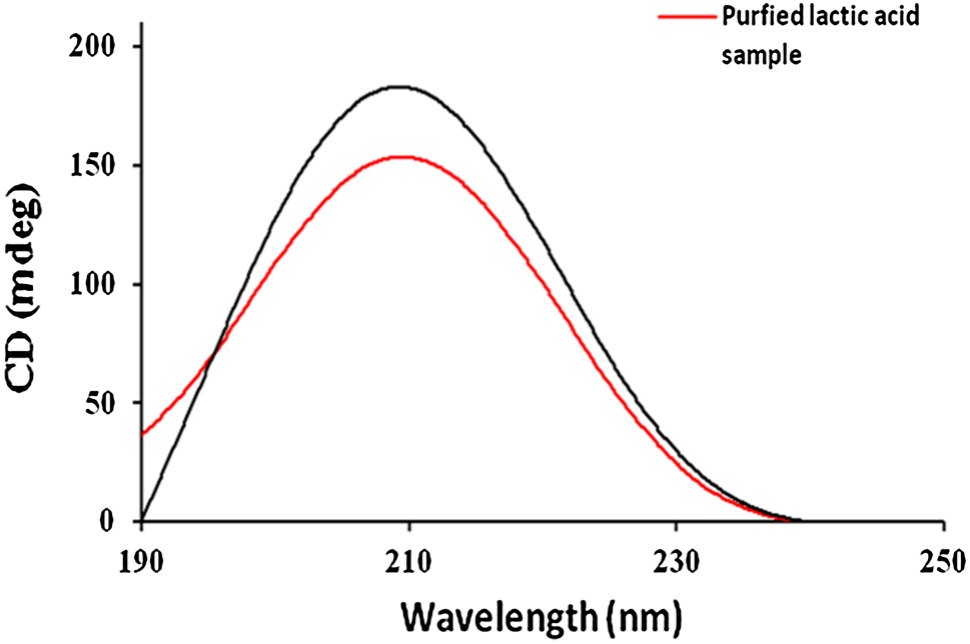
CD spectrum of the purified lactic acid sample along with the pure sigma-graded l-lactic acid where *red line* represents the curve of purified lactic acid sample and *black line* represents the curve of pure sigma-graded l-lactic acid

The stereospecificity analyzed through CD spectropolarimeter was found to be similar to the standard l-lactic acid. It showed band near 210 nm. This absorption band is for α-hydroxy acids. As the degree of rotation is on the positive side which is about 180°, it confirms the levo rotation of the sample. Circular dichroism spectroscopic graph of lactic acid has been shown in Fig. 8.

## Discussion

The study illustrated the platform methodology of purification of lactic acid using fermented broth obtained from *Zizyphus oenophlia*. While designing the purification protocol, several interesting aspects of the process were considered, based on which studies were conducted for optimization of different condition to enhance its purity percentage. Characterization of the same purified sample was carried out to assess the feasibility of the process.

The fermented broth of lactic acid obtained from *Zizyphus* contains various impurities ranging from significant amount of other low-molecular weight carboxylic acids, amino acids, polyphenols, larger proteinaceous molecules, alcohols, esters, metals, traces of sugars, unreacted carbohydrates salts, pigments, DNA, RNA and nutrients along with solid cell biomass (Groot et al. 2010). Depending on the final product specifications, the partial or total elimination of other residual ions (sulfate, phosphate) and remaining sugars (lactose, glucose) is often needed. Hence, based on the nature of contaminants, subsequent purification steps have been chosen. After initial centrifugation and ultrafiltration, a purity of 56.24 % demanded further purification.

Ion exchange chromatography is the reversible adsorption of charged molecules to ion groups of opposite charge immobilized on an inert matrix. At lower pH, the resin showed weaker affinity with the lactic acid. Probable reason of reduced binding at lower pH might be the drastic differences in pH between the resin and the sample (Tong et al. 2004). The affinity of lactic acid seemed to improve with increasing pH. However, pH above 5 showed negative effect on lactic acid binding, which can be attributed to the fact that increase in the negativity of peptides, amino acids (pI within the range of pH 5–7) and phenolics with the increasing pH might have caused decrease in competitive ability of lactic acid binding with the resin. Tong et al. (2004) also showed similar effect within pH 6–7. Another reason for which the adsorption of lactic acid is strongly affected by the initial pH of the sample can be due to the effect of pH on the equilibrium of the undissociated and dissociated acid forms (Moldes et al. 2001). Therefore, the adsorbent that will maximize the recovery of lactic acid is expected to depend on the processing pH as well as on the adsorbent’s basicity (Moldes et al. 2003; Dechow 1989; Evangelista et al. 1994). In this regard, it has also been noticed that, with pKa of lactic acid being 3.85, below this pH no binding has been observed with the resin.

Amberlite IRA-96, by nature, is a weak anion exchanger. It was selected, since it does not split off amines and is important in pharmaceutical and food applications. Moreover, they are easy to regenerate, obtained in fairly pure form, and provide high recovery yield of lactic acid as compared to other resins by Chendake and Kharul (2014). The anion exchange resin Amberlite IRA 96 resins has a macroreticular structure and has maximum porosity that is higher than that of their respective gel-matrix resins. Binding of lactate ion to the ion exchange matrix predominantly occurs following the equation given below:

$${\text{R}}^{ + } {\text{Cl}}^{ - } + {\text{L}}^{ - } \to {\text{R}}^{ + } {\text{L}}^{ - } + {\text{Cl}}^{ - }$$

where L denotes the lactate ion and R the overall stationary matrix containing functional group. This weak base Cl^−^ containing matrix was further exchanged by the counter ions, i.e., lactate ions present in the mobile phase. Initially upon regeneration, R^+^ and Cl^−^ remain in equilibrium within the column. As the counter charge, i.e., lactate anion is introduced in the system, the equilibrium gets shifted and the targeted ion undergoes adsorption through binding reversibly to the matrix with a simultaneous charge displacement of the counter ions. The overall reaction is dependent on relative affinities of the ions, concentration of the ions in solution and the pH of the solution. It was observed that maximum 200 mg lactic acid/g resin got adsorbed. The results obtained showed slightly higher binding capacity of Amberlite IR 96 than that of Amberlite IR 67 as studied earlier (Chendake and Kharul (2014); Kulprathipanja 1991; Kulprathipanja and Oroskar 1991). Also, resins loaded with Cl^−^ form during regeneration reduced the overall steps of lactic acid recovery, thus incurring the cost. Moldes et al. (2001, 2003) have compared four different types of Amberlite resins and finally concluded that IR 96 and 67 are weak base resins having higher binding capacity.

Generally, rapid ion exchange process with high affinity between the resin and the targeted molecules lead to steeper breakthrough curve. Comparative analysis of the slopes at different pH indicated an enhancement in adsorption efficiency of resin with increase in pH. It essentially suggested that the relative affinity of lactic acid towards Amberlite IRA 96 is higher at pH 5 as compared to the same at pH 4, which is also in agreement with the results obtained in pH binding studies. The remaining curves showed a stable lactic acid concentration due to saturation in the effluent (Cao et al. 2002; Kulprathipanja and Oroskar 1991).

Dielectric constant of solvent molecules used during elution plays an important role on the interaction between the eluate and the fixed ionic groups of the resin. It is recommended to use HCl as the eluent because removal of the traces of HCl present in the final solution of lactic acid can easily be achieved owing to its relative volatility and the final product could be obtained in the form of lactic acid only rather than its corresponding salt. It is a well-known fact that retention or elution of ions adsorbed on beads is highly dependent on ionic strength or pH of the eluent. Hence, it might be possible that lower ionic strength (0.1 N) of HCl has enhanced the retention of the contaminating anions on the resin, as lesser number of counter ions has to compete with the analyte (Subramanian 1995; Deyl 1984). The elution was effective at this condition because selective replacement of lactate ions from the beads by Cl^−^ ion might be preferred at that particular ionic strength of HCl. Decrease in purity percentage attributed to the release of other contaminating anionic groups from the beads by higher strength of HCl. This step is supposed to remove all the uncharged and basic (sugar residues, alcohols, amino acids, etc.) components resulting in almost 93 % purity.

Amberlite IR-120, a strongly acidic gel resin which remains in H^+^ form, has been showed to possess highest values of the exchange capacity by many researchers (Madzingaidzo et al. 2002; Quitero et al. 2012). It seemed to be the most promising candidate for carrying out the process. In this case, the washing capacity of the resin was determined at different pH of the input sample and the results showed that, at pH 4 the maximum purity of the sample corresponds to more than 99.17 %. Since, the elution pH was higher than p*K*a value of lactic acid as well as lower than that of amino acids and phenolic acids, lactic acid might have been eluted undisturbed while the contaminants interacted with the matrix resulting in their adsorption.

The band observed in the UV/VIS spectra is the weak π* ← *n* transition of the carboxylic group of lactic acid. A discreet zone observed around 260 nm is a signature of carboxylic group. Similar observation was reported by Myhre and Nielsen (2004). Earlier study conducted by Maria and McGlynn (1972) also showed similar trend for different organic acids. This property of absorbing light below 300 nm wavelength was used for the determination of carboxylic group.

This absorption band at 210 nm is for α-hydroxy acids. Similar kind of bands of CD spectra of lactic acid was reported earlier by Losada and Xu (2007) and Anand and Hargrea (1967) where studies were performed with different α-hydroxy acids in different solvents. Results showed strong positive cotton effect at about 210 nm.

A comparative data for purification of lactic acid reported by different authors have been tabulated in Table 1 from which it can be clearly depicted that the recovery of the present study showed an improvement on the basis of purity and recovery. The improved recovery of lactic acid has a prolonged effect on the overall economy as far as the large-scale application is concerned. Hence, present strategy confirms that a system of column packed with Amberlite IRA-96 and IR-120 could be a suitable technology for purification of fermented broth lactic acid for its application in different industries.

**Table 1:** Comparison of the present results with the reported data on purifications for lactic acid using different ion exchange resins

Ion exchangers used	Type of ion exchanger	Lactic acid purity and recovery yield	References
Amberlite IRA 400	Strong anion exchange resin	94 % purity	Raya-Tonetti et al. (1999)
Amberlite IRA 92	Weak anion exchange resin	Yield of 82.6 % having purity 96.2 %	Tong et al. (2004)
Amberlite IR 120	Strong cation exchange resin	LACTIC acid concentration of 40 g/dm^3^	Rincon et al. (2007)
Amberlite IRA 67	Weak anion exchange resin	Recovery of 92.5–98.67 % of lactic acid	John et al. (2008)
Amberlite IRA-400 and IR-120	Strong anion and strong cation exchange resin	Final lactic acid recovery yield was 73 %	Quintero et al. (2012)
Amberlite IRA-96 and IR-120	Weak anion and strong cation exchange resin	Final lactic acid recovery yield was 96 % of purity more than 99 %	Present study

Hence, the present study delved into the two-step purification process using chromatographic system and the feasibility of this process for the recovery of lactic acid was investigated. Maximum binding capacity was found to 210.46 mg lactic acid/g bead. The other parameters like binding pH, HCl normality as eluent and loading pH for cation exchange process were found to be 7, 0.1 N, 4, respectively. With this simple two-step purification process, lactic acid of 99 % purity was obtained. Thus, the present method of purification can be beneficial in three ways. Firstly, it provided effective means of waste reduction released from other purification processes. Secondly, straightforward downstream purification process was developed which could be economically adopted and scaled up by lactic acid industries. Finally, more than 99 % pure lactic acid has been obtained which opens up frontier for its multitude application. Its characterization validated the formation of only levo rotatory form of lactic acid whose easy metabolism by the human system triggers its application towards biomaterial sector.
